# Dual-Responsive Hybrid Microgels Enabling Phase Inversion in Pickering Emulsions

**DOI:** 10.3390/polym17202762

**Published:** 2025-10-15

**Authors:** Minyue Shen, Lin Qi, Li Zhang, Panfei Ma, Wei Liu, To Ngai, Hang Jiang

**Affiliations:** 1 The Key Laboratory of Synthetic and Biological Colloids, Ministry of Education & School of Chemical and Material Engineering, Jiangnan University, Wuxi 214122, China; 2 Department of Chemistry, The Chinese University of Hong Kong, Shatin, N. T., Hong Kong 999077, China

**Keywords:** hybrid microgels, dual stimuli responsiveness, Pickering emulsions, reversible phase inversion, sol–gel synthesis

## Abstract

Pickering emulsions have emerged as promising multiphase systems owing to their high stability and diverse applications in materials and chemical engineering. However, achieving precise and stimuli-responsive regulation of emulsion type, particularly reversible phase inversion between oil-in-water and water-in-oil states under fixed formulation without additional stabilizers, remains a considerable challenge. In this work, we developed a sol–gel strategy, i.e., in situ hydrolysis and condensation of silane precursors to form a silica shell directly on responsive microgels, to produce H-SiO_2_@P(NIPAM-co-MAA) hybrid microgels. The resulting hybrid particles simultaneously retained pH and temperature responsiveness, enabling the transfer of these properties from the polymeric network to the emulsion interface. When employed as stabilizers, the hybrid microgels allowed the controlled formation of Pickering emulsions that remained stable for one week under testing conditions. More importantly, they facilitated in situ reversible phase inversion under external stimuli. Overall, this work establishes a sol–gel approach to fabricate organic–inorganic hybrid microgels with well-defined dispersion and uniform silica deposition, while preserving dual responsiveness and enabling controlled phase inversion of Pickering emulsions.

## 1. Introduction

Pickering emulsions were first reported by Ramsden in 1903 and Pickering in 1907 [1,2]. Unlike conventional emulsions stabilized by surfactants, Pickering emulsions rely on the irreversible adsorption of solid particles, typically at the nano- or microscale, at the oil–water interface. These particles form a dense physical barrier that effectively suppresses droplet coalescence, thereby imparting superior stability, often allowing emulsions to remain stable for weeks or even months [3,4,5,6]. In addition, Pickering emulsions offer advantages that are difficult to achieve with surfactant-based systems, including cost-effectiveness, recyclability, and enhanced resistance to deformation [7,8].

To achieve tunable emulsification, stimuli-responsive particles have been widely introduced as stabilizers [9,10,11]. Among them, microgels—representative soft colloids—have attracted particular interest due to their reversible swelling behavior. For instance, poly(N-isopropylacrylamide) (PNIPAM) microgels exhibit a volume phase transition temperature (VPTT) near 32 °C, where their network collapses or swells in response to temperature changes [12,13]. Furthermore, functional groups can be incorporated either during synthesis or by post-modification, endowing microgels with additional responsiveness to pH, light, glucose, and other stimuli [14,15]. PNIAPM-based microgels have also been widely investigated in drug delivery, tissue engineering, and biosensors due to their generally acceptable biocompatibility [16,17], although their eco-friendliness and long-term health safety remain under discussion.

Previous studies have demonstrated that microgels can spontaneously adsorb at oil–water interfaces, reduce interfacial tension, and enhance interfacial viscoelasticity [4,18,19]. When employed as stabilizers, responsive microgels can transfer their volume phase transition behavior to emulsions, enabling the regulation of emulsion type and stability [20,21]. Nevertheless, most microgel-stabilized Pickering emulsions are limited to switching between emulsification and demulsification, while extending their regulation to processes such as phase inversion remains a major challenge, since it requires particles to redistribute between oil and water phases despite their strong, irreversible adsorption. At the same time, controllable phase inversion is of considerable importance, as it preserves the emulsion structure while enabling switching between states, offering unique opportunities for tunable release, texture modulation, and interfacial catalysis [22,23]. In addition, research on tailoring the surface properties of microgels is relatively scarce, which restricts their broader application in stimuli-responsive Pickering emulsions. For example, due to their intrinsic hydrophilicity, microgels generally favor the stabilization of oil-in-water (O/W) emulsions, whereas their ability to stabilize water-in-oil (W/O) systems is limited [24].

To address this issue, several strategies have been investigated, including combining microgels with hydrophobic particles to synergistically stabilize W/O emulsions, chemically incorporating hydrophobic moieties into the microgel network, and hybridizing microgels with inorganic colloids [25,26,27,28,29]. Among these, silica–microgel composites have attracted particular attention, with methods such as in situ polymerization or grafting, emulsion templating, and noncovalent self-assembly being reported [30,31,32,33]. For example, Jiang et al. [32] proposed a reverse Pickering emulsion templating method, in which hydrophobic SiO_2_ nanoparticles were deposited in situ on P(NIPAM-co-MAA) microgels within a W/O Pickering emulsion. While this approach successfully produced dual-responsive hybrid microgels, intrinsic drawbacks of the templating strategy include the limited tunability of surface wettability, heterogeneity in size and shell thickness, and sensitivity of shell stability to the degree of crosslinking. Watanabe et al. [34] developed hydrophobized nanocomposite hydrogel microspheres via a seeded emulsion polymerization approach.

Motivated by these challenges, we developed a sol–gel strategy to prepare H-SiO_2_@P(NIPAM-co-MAA) hybrid microgels by in situ growth of silica on dual-responsive P(NIPAM-co-MAA) microgels. By varying the amount of TEOS precursor, hybrid particles with different silica loadings were obtained, while retaining the intrinsic pH and temperature responsiveness of the parent microgels. The sol–gel process offers distinct advantages over physical mixing, in situ precipitation, or templating methods, as it enables more uniform and stable silica deposition under mild conditions and provides covalent anchoring for enhanced structural integrity. The detailed evaluation of their emulsification performance and phase inversion behavior is presented in the following sections.

## 2. Materials and Methods

### 2.1. Materials

Tetraethyl orthosilicate (TEOS, ≥99%) was purchased from Sigma–Aldrich (Shanghai, China). N-isopropylacrylamide (NIPAM, 98%) was obtained from TCI (Shanghai, China). N,N′-Methylenebisacrylamide (BIS, ≥99%), sodium fluorescein (reagent grade), and isobutyltrimethoxysilane (IBTMS, 97%) were supplied by Aladdin Reagent Co., Ltd. (Shanghai, China). γ-Methacryloxypropyltrimethoxysilane (KH-570, 98%), potassium persulfate (KPS, reagent grade), toluene (analytical grade), and methacrylic acid (MAA, reagent grade) were purchased from Sinopharm Chemical Reagent Co., Ltd. (Shanghai, China). Aqueous ammonia solution (25–28%) was obtained from Macklin Biochemical Co., Ltd. (Shanghai, China), and anhydrous ethanol (analytical grade) was purchased from Titan Technology Co., Ltd. (Shanghai, China). PolyFluor^®^570 (reagent grade) was obtained from Polysciences, Inc. (Warrington, PA, USA). All chemicals were used as received without further purification.

### 2.2. Synthesis of P(NIPAM-co-MAA) Microgels

P(NIPAM-co-MAA) microgels were synthesized by precipitation polymerization. NIPAM (2 g), MAA (500 μL), BIS (0.06 g), and PolyFluor^®^570 solution (500 μL, 1 mg/mL in 10% *v*/*v* ethanol–water) were dissolved in 150 mL of deionized water in a 250 mL three-necked flask under ultrasonication. The solution was preheated to 45 °C in a water bath and deoxygenated by nitrogen bubbling for 45 min with continuous stirring. The temperature was then increased to 70 °C while maintaining nitrogen flow. KPS (0.12 g) dissolved in 5 mL of deionized water was injected into the flask to initiate polymerization. Within several minutes, the solution turned opalescent, a phenomenon commonly observed during colloidal particle nucleation in precipitation polymerization [35]. After 1 h, γ-Methacryloxypropyltrimethoxysilane (KH-570, 320 μL) was introduced as a silane coupling agent to provide reactive sites for subsequent silica grafting. The reaction proceeded for an additional 5 h. The resulting dispersion was purified by centrifugation at 8000 rpm for 10 min (Multifuge X1R, Thermo Fisher Scientific, Waltham, MA, USA), redispersion in deionized water, and repeated for four centrifugation–redispersion cycles. The final purified product was stored as an aqueous dispersion.

### 2.3. Synthesis of H-SiO_2_@P(NIPAM-co-MAA) Hybrid Microgels

The sol–gel process was employed to grow silica shells on the surface of P(NIPAM-co-MAA) microgels. Briefly, 30 mL of purified P(NIPAM-co-MAA) aqueous dispersion (Section 2.2) was mixed with 120 mL of anhydrous ethanol and 6 mL of aqueous ammonia (25–28%) in a 250 mL round-bottom flask under vigorous stirring. Subsequently, 15 mL of TEOS dissolved in ethanol–water mixtures of varied concentrations was added dropwise into the system at a constant rate of 12 mL/h. After complete addition, the reaction was maintained for 2 h to allow silica growth. IBTMS was further introduced to tailor the surface properties of the silica shell. The modification reaction was continued for 3 h. Different TEOS and IBTMS dosages were employed to tune the silica loading on the H-SiO_2_@P(NIPAM-co-MAA) hybrid particle, as summarized in Table 1. Apart from these differences in dosage, all samples (S1–S3) were prepared using the same sol–gel protocol under identical conditions.

After reaction, the dispersion was centrifuged at 5000 rpm for 10 min, and the supernatant was decanted. The pellet was redispersed in an ethanol–toluene mixture and centrifuged again; this centrifugation–redispersion step was repeated five times. The final pellet was redispersed in toluene for storage.

To obtain hybrid microgels in dry form, the samples were purified through a solvent-exchange process prior to lyophilization. Specifically, the dispersion was sequentially washed and redispersed in anhydrous ethanol, 70% ethanol–water, 30% ethanol–water, 10% ethanol–water, and finally deionized water. At each step, centrifugation was performed at 5000 rpm for 10 min, followed by redispersion in the next solvent. After the final wash, the purified pellet was redispersed in deionized water and freeze-dried to obtain hybrid microgels as dry powders.

### 2.4. Preparation of Pickering Emulsions

Pickering emulsions were prepared by mixing 2 wt% dispersions of H-SiO_2_@P(NIPAM-co-MAA) hybrid microgels in toluene with deionized water. The mixtures were vortexed for 30 s to obtain emulsions, a duration sufficient to produce stable Pickering emulsions without excess shear. To systematically investigate the influence of formulation and external conditions, emulsions were prepared with oil fractions ranging from 50 to 66.6 vol% (nine increments), at pH values of 2, 4, 6, 8, 10, and 12, and at temperatures of 4, 25, 35, and 50 °C. The emulsion type was determined by the drop volume method combined with microscopic observation.

### 2.5. In Situ Reversible Phase Inversion

Emulsions were first prepared at pH 7 and 50 °C by mixing equal volumes of H-SiO_2_@P(NIPAM-co-MAA) toluene dispersion (2 wt%) and deionized water, followed by vortexing for 30 s. The pH of the emulsion was then adjusted to 2 by adding 1 M HCl solution, and the mixture was vortexed again at 50 °C. Subsequently, the emulsion was cooled to 4 °C, equilibrated, and vortexed once more. At each stage, the emulsion type was examined using the drop volume method and microscopic observation.

### 2.6. Characterization

#### 2.6.1. Scanning Electron Microscopy (SEM)

The morphology of microgels and hybrid microgels was examined by field-emission scanning electron microscopy (FE-SEM, S-4800, Hitachi, Tokyo, Japan) operated at an accelerating voltage of 3 kV and a probe current of 10 μA. Dilute dispersions (0.01 wt%) were deposited onto cleaned silicon wafers, dried at room temperature, fixed on conductive tape, and sputter-coated with gold. For pH- or temperature-dependent observations, dispersions were treated under the respective conditions prior to sample preparation.

#### 2.6.2. Dynamic Light Scattering (DLS)

Hydrodynamic diameters were measured by dynamic light scattering using a particle size analyzer (ZEN3700, Brookhaven Instruments Corp., Holtsville, NY, USA) equipped with a 640 nm laser at a fixed scattering angle of 90°. Dispersions (3 mL, 1 × 10^−4^ wt%) were transferred to disposable plastic cuvettes for measurement. P(NIPAM-co-MAA) microgels and H-SiO_2_@P(NIPAM-co-MAA) hybrid microgels were dispersed in deionized water, with solvent refractive index (1.333) and viscosity (0.8872 mPa·s) set to those of water at 25 °C. For pH-responsive measurements, samples were prepared at the designated pH values; for temperature-responsive measurements, data were collected from 22 to 42 °C in 2 °C increments using the instrument’s built-in temperature control. The instrument’s built-in correction for the viscosity of water at each temperature was applied. Each acquisition lasted 2 min, and each sample was measured in triplicate.

#### 2.6.3. Zeta Potential

Zeta potentials were determined by electrophoretic light scattering on the same instrument (ZEN3700, Brookhaven Instruments Corp., Holtsville, NY, USA). Dispersions were diluted to 0.01 wt%, and pH was adjusted with 1 M HCl or 1 M NaOH prior to measurement. Samples were loaded into disposable plastic cuvettes with inserted electrodes according to the manufacturer’s instructions. For each condition, three independent measurements were performed.

#### 2.6.4. Thermogravimetric Analysis (TGA)

TGA was performed on a TGA/1100SF instrument (Mettler Toledo, Greifensee, Switzerland) to determine the SiO_2_ content of hybrid microgels with different surface loadings. Approximately 5–10 mg of each sample was placed in an alumina crucible and heated from 100 to 800 °C at a constant heating rate of 10 K/min under an air flow of 200 mL/min. The inorganic residue remaining at 800 °C was taken as the silica content.

#### 2.6.5. Contact Angle Measurements

Air–water contact angles were determined using a video-based optical contact angle goniometer (OCA15EC, DataPhysics Instruments GmbH, Filderstadt, Germany) operated in the sessile drop mode. Thin films were prepared by drop-casting 3 wt% dispersions of microgels or hybrid microgels onto cleaned silicon wafers, followed by drying at room temperature to form uniform coatings. Water droplets (2.5 μL) were deposited on the film surface using a microsyringe, and the contact angles were recorded by the instrument’s imaging system. For pH- or temperature-dependent measurements, samples were pretreated under the corresponding conditions prior to film preparation. Each measurement was repeated three times to ensure reproducibility.

#### 2.6.6. Dynamic Interfacial Tension (IFT) Measurements

IFT was measured using the pendant drop method on the same instrument (OCA15EC, DataPhysics Instruments GmbH, Filderstadt, Germany). For P(NIPAM-co-MAA) microgels, 10 μL of aqueous dispersions were injected through a needle into the toluene phase to form pendant droplets. For H-SiO_2_@P(NIPAM-co-MAA) hybrid microgels, 10 μL of toluene dispersions were injected into the aqueous phase in the same manner. The time-dependent interfacial tension was recorded in real time at a frequency of 1 frame per second (fps) by the instrument’s imaging system.

#### 2.6.7. Optical Microscopy

The morphology and microstructure of Pickering emulsions were examined by optical microscopy (Ni-U, Nikon, Tokyo, Japan). For observation, a defined volume of the continuous phase was first added into a concave cavity slide, followed by the addition of an appropriate amount of Pickering emulsion. Droplet structures were then imaged, and interfacial features were recorded using a 100× oil-immersion objective.

#### 2.6.8. Confocal Laser Scanning Microscopy (CLSM)

CLSM (Nikon AX, Nikon, Tokyo, Japan) was used to examine the type and interfacial microstructure of O/W and W/O Pickering emulsions stabilized by hybrid microgels. Samples were mounted in fluorinated silicone spacers with concave cavities and sealed with coverslips to minimize evaporation of the continuous phase. Fluorescence images were acquired at a resolution of 1024 × 1024 pixels using excitation wavelengths of 405 nm (blue), 488 nm (green), and 561 nm (red). Interfacial structures were recorded with a 100× oil-immersion objective.

#### 2.6.9. Droplet Size and Shape Analysis

CLSM images (single focal plane) were analyzed using ImageJ software version 1.54f (National Institutes of Health, Bethesda, MD, USA). Droplet boundaries were segmented, and the following parameters were extracted:

- Equivalent circular diameter (ECD, μm): calculated from the projected droplet area,

(1) $$ECD=\sqrt{4A/\pi };$$

- Aspect ratio (AR): obtained from ellipse fitting, defined as major/minor axis length;
- Standard deviation (SD): statistical spread of ECD and AR within each sample set;
- Coefficient of variation (CV): defined as SD/mean, a dimensionless measure of polydispersity.

At least 50 droplets were analyzed per condition to ensure statistical reliability.

## 3. Results and Discussion

### 3.1. Structure and Characterization of H-SiO_2_@P(NIPAM-co-MAA) Hybrid Microgels

**Figure 1:**
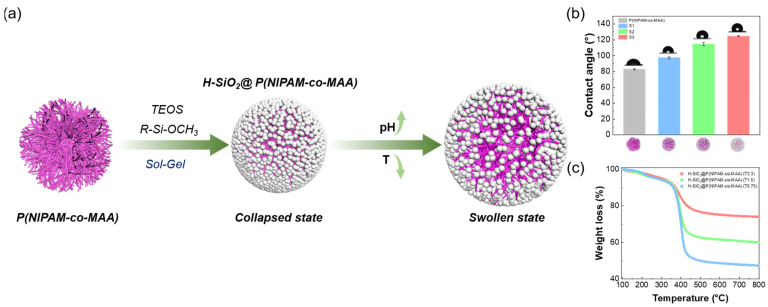
Morphological and compositional characterization of P(NIPAM-co-MAA) microgels and H-SiO_2_@P(NIPAM-co-MAA) hybrid microgels. (**a**) Schematic illustration of the two-step synthesis of H-SiO_2_@P(NIPAM-co-MAA) hybrid microgels; (**b**) air-water contact angles and (**c**) TGA curves of the hybrid microgels. Scale bar in (**b**): 1 μm.

The preparation of H-SiO_2_@P(NIPAM-co-MAA) hybrid microgels involved a two-step process (Figure 1a). First, P(NIPAM-co-MAA) microgels were synthesized by precipitation polymerization, during which KH-570 was introduced to provide reactive sites for subsequent silica growth. In the second step, TEOS and IBTMS were added to the dispersion of P(NIPAM-co-MAA). A sol–gel reaction promoted the in situ deposition of silica on the microgel surface, yielding hydrophobic H-SiO_2_@P(NIPAM-co-MAA) hybrid microgels. Since P(NIPAM-co-MAA) exhibits dual pH and temperature responsiveness [36], the hybrid products were expected to retain similar stimuli-responsive characteristics.

To confirm the stimuli-responsive properties, the morphology of P(NIPAM-co-MAA) microgels under different pH and temperature conditions was examined by SEM. As shown in Figure S1, microgels remained collapsed with nearly spherical morphology at acidic pH values (pH 2 and 4), with an average diameter of approximately 270 nm. At pH 6, the particles swelled significantly, adopting a flattened, faceted morphology with concave inter-facet regions, with a diameter of ~410 nm. Further increasing the pH to 10 resulted in a slight additional increase to ~425 nm. This pH responsiveness arises from the deprotonation of –COOH groups in alkaline conditions, generating –COO^−^ moieties that induce electrostatic repulsion between polymer chains and promote water uptake, while in acidic conditions protonation reduces repulsion and leads to network collapse [37].

Temperature responsiveness was also confirmed by SEM images (Figure S2). At 50 °C, microgels were in a collapsed state with an average diameter of ~255 nm, whereas at 4 °C, particles swelled to ~392 nm. These results demonstrate that P(NIPAM-co-MAA) exhibits the typical thermo-responsive behavior of PNIPAM, driven by hydrogen-bonding interactions between amide groups and water molecules, consistent with previous reports on PNIPAM-based microgels [38].

**Figure 2:**
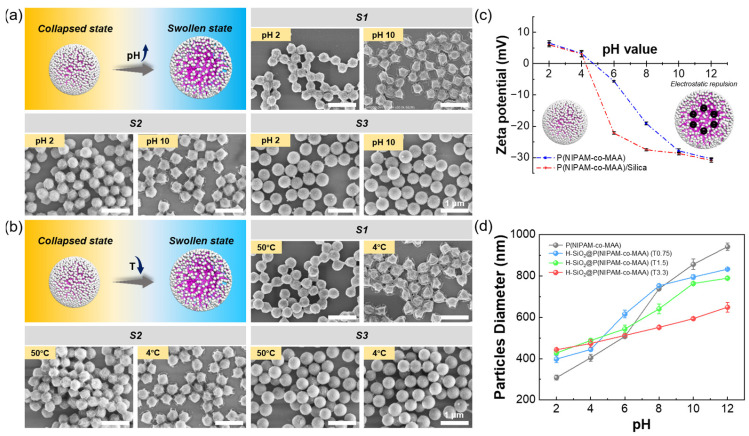
pH and temperature responsiveness of P(NIPAM-co-MAA) microgels and H-SiO_2_@P(NIPAM-co-MAA) hybrid microgels. (**a**) SEM images of hybrid microgels (S1, S2, S3) at pH 2 and pH 10; (**b**) SEM images of hybrid microgels at 4 °C and 50 °C; (**c**) zeta potential and (**d**) hydrodynamic diameters of P(NIPAM-co-MAA) microgels and hybrid microgels at various pH values. Scale bar in (**a**,**b**): 1 μm.

After establishing the dual responsiveness of P(NIPAM-co-MAA), hybrid microgels with varying silica loading were obtained by adjusting the TEOS dosage (0.75, 1.5, and 3.3 mL), corresponding to samples S1, S2, and S3, respectively (Table 1, Figure 2a). SEM images revealed that under basic conditions (pH 10), the morphology evolved from flattened structures to spherical particles with increasing TEOS dosage. At 0.75 mL TEOS (S1), only sparse silica nanoparticles were observed on the surface. At 1.5 mL (S2), the microgels exhibited a more spherical morphology, while at 3.3 mL (S3), they were fully encapsulated by a dense silica shell, forming rigid spherical particles. Quantitative analysis of SEM images (measured by maximum particle dimension) gave average diameters of approximately 441 nm (S1), 438 nm (S2), and 471 nm (S3). These findings indicate that the morphology of H-SiO_2_@P(NIPAM-co-MAA) can be effectively tuned by the TEOS dosage.

In addition, contact angle measurements demonstrated a direct correlation between the silica loading on the particle surface and the surface wettability of the hybrid microgels (Figure 1b). At lower TEOS dosages, the silica coverage was limited, leaving partial hydrophilic groups exposed. As a result, the hybrid microgels exhibited higher hydrophobicity compared to pure P(NIPAM-co-MAA), but still lower than those with complete silica encapsulation. With increasing TEOS dosage, the microgel surfaces became progressively covered by denser silica layers. For instance, sample S3 was almost fully encapsulated by silica, leading to markedly enhanced hydrophobicity.

Thermogravimetric analysis (TGA) was further employed to quantify the silica content (Figure 1c). The results showed that silica loading increased from 47.3% in S1 to 74.0% in S3 with increasing TEOS dosage, confirming that the inorganic component was predominantly deposited on the microgel surface and that its proportion rose substantially with the amount of precursor used.

### 3.2. pH and Temperature Responsiveness of H-SiO_2_@P(NIPAM-co-MAA) Hybrid Microgels

Evaluating whether H-SiO_2_@P(NIPAM-co-MAA) hybrid microgels retain the dual responsiveness of the parent P(NIPAM-co-MAA) microgels is essential for confirming their suitability as stimuli-responsive stabilizers for Pickering emulsions. Similar to the hydrogen-bond-dependent volume phase transition of thermo-responsive microgels, the pH-dependent response originates from the ionization state of surface functional groups. Changes in ionization alter the surface charge density and zeta potential, thereby modulating electrostatic repulsion among polymer chains and leading to either swelling or collapse in aqueous media. To verify this behavior, the zeta potentials of P(NIPAM-co-MAA) and H-SiO_2_@P(NIPAM-co-MAA) were measured under different pH conditions. As shown in Figure 2c, P(NIPAM-co-MAA) was positively charged at pH 2 (ζ = +6.54 ± 0.77 mV), but its charge shifted to negative values with increasing pH (ζ = −30.43 ± 0.36 mV at pH 12) due to deprotonation of –COOH groups to –COO^−^. The isoelectric point was located between pH 4 and 5 (ζ = +3.25 ± 1.02 mV at pH 4; −5.62 ± 0.25 mV at pH 6), corresponding to the onset of the volume phase transition. A similar trend was observed for the hybrid microgels (ζ = +6.01 ± 0.69 mV at pH 2; −30.91 ± 0.57 mV at pH 12), with the isoelectric point also located between pH 4 and 5 (ζ = +3.10 ± 0.91 mV at pH 4; −22.19 ± 0.37 mV at pH 6), suggesting that these materials undergo swelling under alkaline conditions due to electrostatic repulsion.

Morphological changes under different stimuli were further examined by SEM to validate the dual-responsive behavior (Figure 2a,b). Samples S1 and S2, which had relatively low silica loadings, exhibited clear pH responsiveness similar to P(NIPAM-co-MAA) (Figure 2a). From pH 2 to 10, the average diameter of S1 increased from approximately 307 nm to 441 nm, while S2 increased from 367 nm to 438 nm. The smaller size change in S2 indicates that higher silica coverage restricted the extent of volume transition. In contrast, sample S3, which was nearly fully encapsulated by silica, displayed negligible size variation across the same pH range. Temperature responsiveness was also observed; S1 and S2 showed significant size differences between 4 °C and 50 °C (Figure 2b), whereas S3 maintained almost unchanged morphologies. These results confirm that the density of the inorganic shell directly influences the magnitude of volume phase transition.

DLS measurements were conducted to evaluate the swelling behavior in dispersion (Figure 2d). Pure P(NIPAM-co-MAA) microgels in water displayed pronounced pH responsiveness, with hydrodynamic diameters increasing from approximately 300 nm at pH 2 to approximately 940 nm at pH 12. The swelling in dispersion was much greater than that observed in SEM due to water permeation into the three-dimensional polymer network. Hybrid microgels S1 and S2 also exhibited pH-dependent size variations of about 400 nm, though the transition amplitude was smaller than that of pure microgels, suggesting that the diminished pH-responsiveness at higher silica coverage is mainly due to the constraining effect of a dense silica shell. While changes in polymer crosslinking density are unlikely in this two-step route (the microgel network was pre-formed with fixed crosslinker content), possible alternations of the internal network structure, such as partial penetration/embedding of silica within the polymer matrix, may also contribute. Notably, even S3, with a fully encapsulated silica layer, retained limited pH responsiveness. Overall, these findings demonstrate that H-SiO_2_@P(NIPAM-co-MAA) hybrid microgels possess both pH and temperature dual responsiveness, although the magnitude of response decreases with increasing silica loading. Their ability to swell in aqueous media suggests that emulsions stabilized by these particles are also likely to exhibit stimuli-responsive behavior.

### 3.3. Responsive Behavior of Pickering Emulsions Stabilized by H-SiO_2_@P(NIPAM-co-MAA) Hybrid Microgels

**Figure 3:**
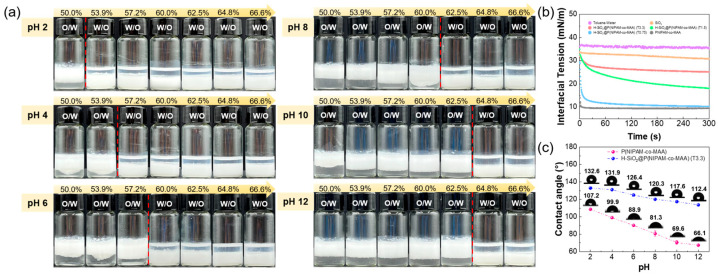
Interfacial activity and pH-responsive phase behavior of hybrid microgels in Pickering emulsions. (**a**) Appearance of Pickering emulsions stabilized by H-SiO_2_@P(NIPAM-co-MAA) (S3) at different oil-water ratios and pH values at 25 °C. The red dashed lines separate the two samples showing emulsions of different types; (**b**) dynamic interfacial tension at the interfaces of water-toluene, SiO_2_ toluene dispersion-water, P(NIPAM-co-MAA) aqueous dispersion-toluene, and H-SiO_2_@P(NIPAM-co-MAA) toluene dispersion–water; (**c**) air–water contact angles of P(NIPAM-co-MAA) and S3 at various pH values.

Microgels possess high interfacial activity and can spontaneously adsorb at oil–water interfaces to reduce interfacial tension, making them effective stabilizers for Pickering emulsions. For H-SiO_2_@P(NIPAM-co-MAA), whether emulsification can be achieved depends on its interfacial activity. To evaluate this, the effect of hybrid microgels with different silica loadings on oil–water interfacial tension was examined. As shown in Figure 3b, the interfacial tension between pure water and toluene was high and remained nearly constant over time in the absence of particles. The addition of silica particles produced no significant reduction, indicating their poor interfacial activity. In contrast, the introduction of P(NIPAM-co-MAA) or H-SiO_2_@P(NIPAM-co-MAA) dispersions led to a pronounced decrease in interfacial tension, with pure P(NIPAM-co-MAA) showing the strongest effect. Increasing silica coverage progressively weakened the interfacial activity of the hybrid microgels. Overall, H-SiO_2_@P(NIPAM-co-MAA) hybrid microgels exhibited sufficient interfacial activity to act as stabilizers for Pickering emulsions.

After confirming the emulsification capability, the responsiveness of the hybrid microgel-stabilized emulsions was further investigated. Figure 3a shows the effects of oil fraction and pH on emulsions stabilized by S3. At constant pH, the system favored O/W emulsions at low oil fractions, while increasing oil fraction induced inversion to W/O emulsions. The critical oil fraction for inversion varied with pH: only 53.9 vol% oil was required at pH 2, whereas 64.8 vol% was necessary at pH 10 or 12. This behavior is attributed to particle wettability: under acidic conditions, the microgels collapsed and became more hydrophobic, favoring W/O emulsions, whereas under alkaline conditions, deprotonation of –COOH groups induced swelling and greater hydrophilicity, promoting O/W emulsions.

To verify this explanation, air–water contact angle measurements of P(NIPAM-co-MAA) and S3 were performed at different pH values (Figure 3c). Both materials exhibited decreasing contact angles with increasing pH. For P(NIPAM-co-MAA), the angle decreased from 107.2° at pH 2 to 66.1° at pH 12, indicating a hydrophobic-to-hydrophilic transition. For S3, the contact angle decreased from 132.6° at pH 2 to 112.4° at pH 12. The decreasing contact angle with increasing pH confirms enhanced hydrophilicity of the hybrid microgels, which is consistent with the observed phase inversion. However, contact angle alone may not fully account for this phenomenon. According to previous studies, phase inversion is generally influenced by a combination of factors, including interfacial energy redistribution and particle reorganization at the oil–water boundary [39,40]. While these parameters were not directly measured in this work, our results are in line with such mechanisms.

**Figure 4:**
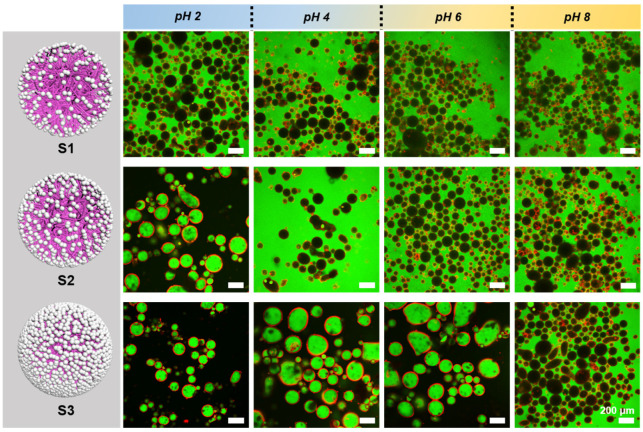
CLSM images of Pickering emulsions stabilized by H-SiO_2_@P(NIPAM-co-MAA) (S1, S2, S3) at different pH values at 25 °C with an oil-water ratio of 3:2. All CLSM images shown represent single focal planes. Scale bar: 200 μm.

The effect of silica loading was further assessed by comparing emulsions stabilized with S1, S2, and S3 (Figure 4). At 25 °C and an oil fraction of 60 vol%, CLSM images revealed distinct emulsion types. Green fluorescence (fluorescein sodium) marked the aqueous phase, while red fluorescence (PolyFluor^®^570) labeled the hybrid microgels, enabling clear identification of emulsion type. Due to the exposure of hydrophilic groups, S1 exclusively formed O/W emulsions under all pH conditions. S2, with higher hydrophobicity, could already stabilize W/O emulsions at pH 2. S3, with its surface almost fully covered by hydrophobic silica, maintained W/O emulsions up to pH 6. Magnified CLSM images and 3D reconstructions (Figure S3) confirmed that hybrid microgels were uniformly adsorbed at droplet interfaces, forming compact barriers that enhance emulsion stability.

Quantitative droplet size statistics are summarized in Table 2. At acidic pH values (2–4), emulsions stabilized by S1–S3 exhibited relatively larger mean droplet diameters (≈100–150 μm) and higher polydispersity (CV ≈ 0.35–0.46). The shape descriptors also indicate more irregular morphology, with AR values deviating from unity (≈1.3–1.4). Upon increasing the pH to neutral and alkaline conditions (6–8), the mean droplet size decreased steadily (≈80–110 μm), accompanied by lower polydispersity (CV < 0.35) and more spherical morphology (AR ≈ 1.2–1.3). The emulsion type, on the other hand, correlated with droplet size distribution and polydispersity. At acidic pH, where W/O emulsions were more predominant, droplets were larger and more polydisperse; in contrast, at alkaline pH, O/W emulsions were stabilized, exhibiting smaller and more monodisperse droplets. The underlying differences may relate to interfacial activity, particle packing efficiency, or even droplet coalescence dynamics, though further investigation would be required to distinguish these effects.

**Figure 5:**
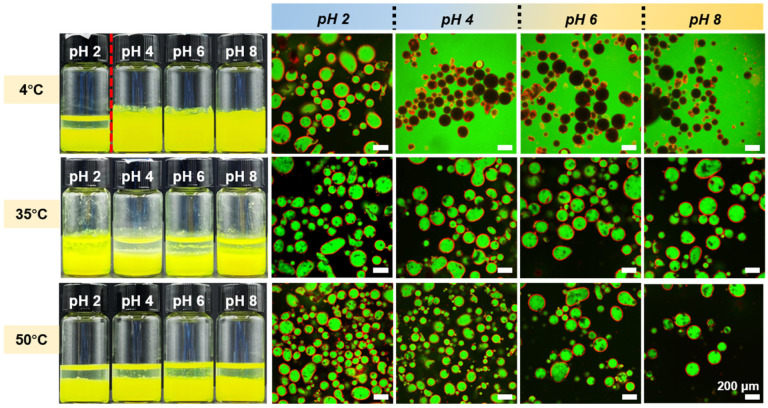
Appearance and CLSM images of Pickering emulsions stabilized by H-SiO_2_@P(NIPAM-co-MAA) (S3) at various temperatures and pH values with an oil-water ratio of 3:2. The red dashed line separates the two samples showing emulsions of different types. All CLSM images shown represent single focal planes. Scale bar: 200 μm.

The dual responsiveness of hybrid microgel-stabilized emulsions to pH and temperature was then investigated. Figure 5 presents the appearance and CLSM images of emulsions prepared with S3 at an oil fraction of 60 vol% under different pH and temperature conditions. Comparisons can be made in two dimensions: horizontally (effect of pH at constant temperature) and vertically (effect of temperature at constant pH). Horizontally, the emulsions exhibited pH responsiveness, shifting from W/O to O/W as pH increased, consistent with the pH-dependent wettability of S3. Vertically, the emulsions exhibited thermal responsiveness: increasing temperature induced inversion from O/W to W/O at constant pH. At 4 °C, hydrogen bonding between amide groups of PNIPAM and water favored hydrophilicity, yielding O/W emulsions except at pH 2. Above the VPTT, these interactions were disrupted, the particles became more hydrophobic, and only W/O emulsions were observed. Collectively, these results confirm that emulsions stabilized by H-SiO_2_@P(NIPAM-co-MAA) are responsive to both pH and temperature, with emulsion type controllable by external stimuli. The full droplet size statistics of S3-stabilized emulsions at different temperatures (4–50 °C) and pH values are provided in Table S1 (Supporting Information).

Finally, the long-term stability of emulsions was evaluated (Figure S4). Emulsions stabilized by S3 at 25 °C, pH 7, and an oil-water ratio of 2:1 were monitored. CLSM images showed that the freshly prepared emulsions were W/O type and remained stable for at least 7 days at room temperature, with no obvious coalescence or internal phase leakage observed. Although the stability was monitored only up to 7 days in this study, the observed performance is consistent with previous reports on responsive or hybrid microgel-stabilized emulsions, which generally exhibit stability windows of several days to a few weeks under similar conditions [41,42].

### 3.4. In Situ Reversible Phase Inversion of Pickering Emulsions

**Figure 6:**
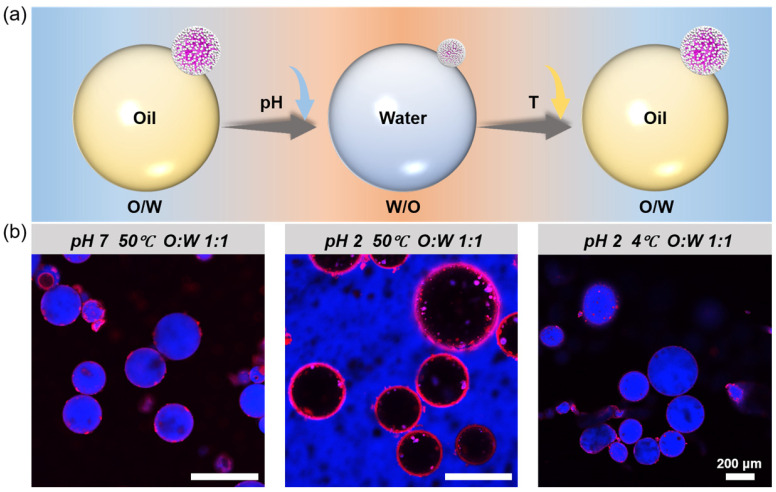
In situ reversible phase inversion of Pickering emulsions under pH and temperature stimuli. (**a**) Schematic illustration of the in situ reversible phase inversion of Pickering emulsions stabilized by H-SiO_2_@P(NIPAM-co-MAA) (S3) under pH and temperature stimuli; (**b**) CLSM images showing the reversible inversion between O/W and W/O emulsions under sequential changes in pH and temperature. All CLSM images shown represent single focal planes. Scale bar in (**b**): 200 μm.

The ability of H-SiO_2_@P(NIPAM-co-MAA)-stabilized Pickering emulsions to undergo in situ reversible phase inversion under dual pH and temperature stimuli was further evaluated (Figure 6). Under the initial condition (pH 7, 50 °C), the hybrid microgels were in a collapsed state, and vortex emulsification yielded O/W-type emulsions. Upon decreasing the pH to acidic conditions, the emulsion inverted to a W/O type; subsequent cooling enabled the system to revert to the O/W type. Notably, throughout this cycle, the dispersed droplets consistently inverted between being the internal and the continuous phase at each step, demonstrating that the hybrid microgels can achieve stable and controllable in situ phase inversion under the combined influence of pH and temperature. These findings highlight the potential of this system for intelligent responsiveness and reversible regulation.

## 4. Conclusions

In this work, H-SiO_2_@P(NIPAM-co-MAA) hybrid microgels with tunable silica loading while retaining dual (pH/temperature) responsiveness were successfully fabricated via a sol–gel strategy. As a stabilizer, these hybrid microgels formed stable Pickering emulsions (tested ≥ 7 days) and enabled in situ droplet-type inversion between O/W and W/O under fixed formulation across the examined pH (2–8) and temperature (4–50 °C) windows. Quantitative analyses of droplet size and polydispersity provided a more rigorous description of the emulsions and revealed consistent trends with silica coverage. The versatility of this approach lies in adjustable silica loading, access to both emulsion types without additional stabilizers, and the operational simplicity of the sol–gel method, which is potentially scalable. Beyond this proof-of-concept, the strategy may be extended to other responsive microgels and oil–water system, with relevance to applications such as personal-care formulations and biphasic catalysis.

## Figures and Tables

**Table 1:** Experimental conditions for H-SiO_2_@P(NIPAM-co-MAA) synthesis at different TEOS and IBTMS dosages.

Sample ID	TEOS/mL	IBTMS/mL	Molar Ratio (TEOS:IBTMS)
S1	0.75	0.225	2.87:1
S2	1.50	0.45	2.86:1
S3	3.30	1.00	2.83:1

Molar amounts: TEOS = 3.36, 6.73, 14.8 mmol; IBTMS = 1.11, 2.23, 4.96 mmol for S1–S3, respectively. Calculated based on the densities and molecular weights of TEOS (ρ = 0.933 g/mL, MW = 208.33 g/mol) and IBTMS (ρ = 0.93 g/mL, MW = 178.30 g/mol).

**Table 2:** Droplet size statistics of emulsions stabilized by S1–S3 hybrid microgels at 25 °C under different pH conditions.

Sample ID	pH	Mean ECD ± SD (μm)	CV	Mean AR ± SD	Type
S1	2	103.3 ± 35.9	0.35	1.28 ± 0.42	O/W
	4	101.8 ± 32.7	0.32	1.21 ± 0.37	O/W
	6	93.4 ± 32.5	0.35	1.28 ± 0.36	O/W
	8	82.1 ± 27.0	0.33	1.22 ± 0.30	O/W
S2	2	129.3 ± 45.4	0.35	1.42 ± 0.54	W/O
	8	104.2 ± 36.2	0.35	1.21 ± 0.22	O/W
	4	83.2 ± 25.4	0.31	1.13 ± 0.21	O/W
	6	87.2 ± 32.9	0.38	1.19 ± 0.34	O/W
S3	2	103.7± 38.4	0.37	1.28 ± 0.40	W/O
	4	141.7 ± 65.5	0.46	1.29 ± 0.26	W/O
	6	160.2 ± 73.3	0.46	1.32 ± 0.44	W/O
	8	99.2 ± 32.5	0.33	1.26 ± 0.34	O/W

## Data Availability

The original contributions presented in this study are included in the article and the Supplementary Material. Further inquiries can be directed to the corresponding authors.

## Supplementary Materials

The following supporting information can be downloaded at: https://www.mdpi.com/article/10.3390/polym17202762/s1, Figure S1: pH-responsive morphology of P(NIPAM-co-MAA) microgels; Figure S2: Thermo-responsive morphology of P(NIPAM-co-MAA) microgels; Figure S3: CLSM characterization of single O/W and W/O emulsion droplets; Figure S4: Stability of W/O Pickering emulsions stabilized by hybrid microgels; Table S1: Droplet size statistics of emulsions stabilized by S3 hybrid microgels at different temperatures (4–50 °C) and pH conditions.
