# Supplementary material for: Dual-Responsive Hybrid Microgels Enabling Phase Inversion in Pickering Emulsions
Source: Polymers (Basel). 2025 Oct 15;17(20):2762. doi: 10.3390/polym17202762 (PMC12566968; doi:10.3390/polym17202762)
Supplement: Supplementary file 1 [file polymers-17-02762-s001.zip › polymers-3897458-supplementary.pdf]

Supplementary

Dual-Responsive Hybrid Microgels Enabling Phase Inversion in Pickering Emulsions

Minyue Shen [1,†], Lin Qi [1,†], Li Zhang [1], Panfei Ma [1], Wei Liu [1], To Ngai [2,*] and Hang Jiang [1,*]

[1] The Key Laboratory of Synthetic and Biological Colloids, Ministry of Education & School of Chemical and Material Engineering, Jiangnan University, Wuxi 214122, China; 6220606020@stu.jiangnan.edu.cn (M.S.); 6210610038@stu.jiangnan.edu.cn (L.Q.); 7220610009@stu.jiangnan.edu.cn (L.Z.); 6230606079@stu.jiangnan.edu.cn (P.M.); weiliu@jiangnan.edu.cn (W.L.)

[2] Department of Chemistry, The Chinese University of Hong Kong, Shatin, N. T., Hong Kong 999077, China

* Correspondence: tongai@cuhk.edu.hk (T.N.); hangjiang@jiangnan.edu.cn(H.J.); †These authors share first authorship.

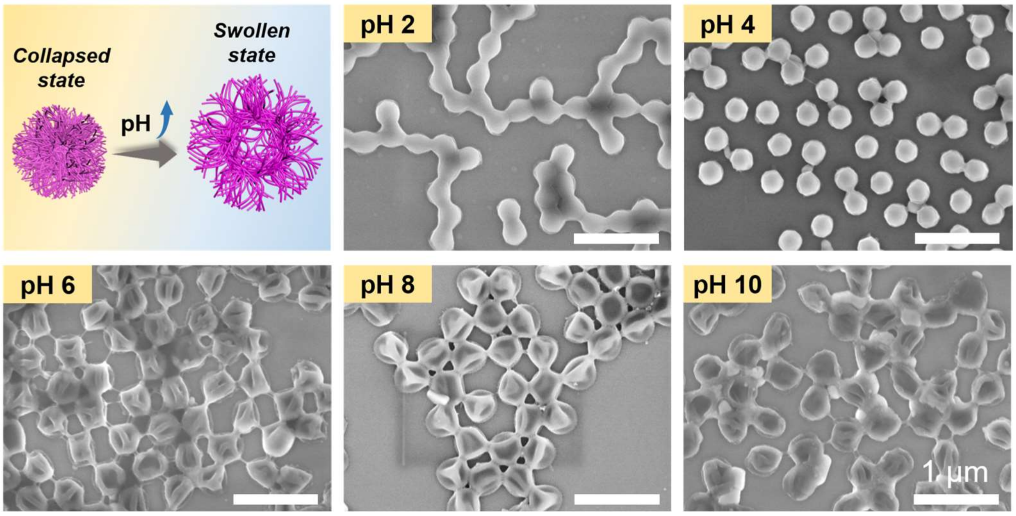

Figure S1. pH-responsive morphology of P(NIPAM-co-MAA) microgels. Scanning electron microscopy (SEM) images of P(NIPAM-co-MAA) microgels at various pH values, illustrating the changes in particle size and morphology induced by pH variation. Scale bar: 1 μm.

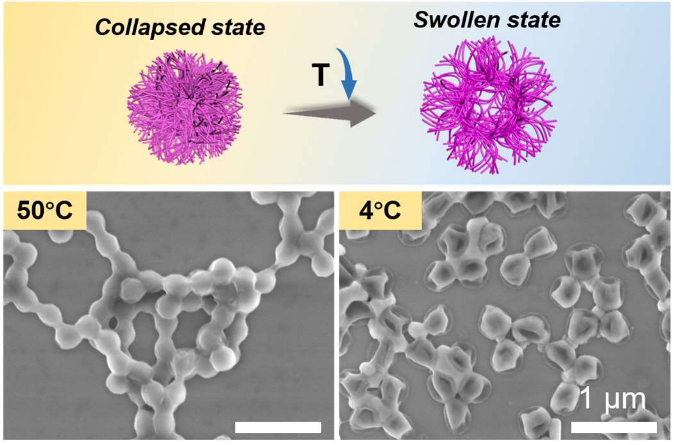

Figure S2. Thermo-responsive morphology of P(NIPAM-co-MAA) microgels. SEM images of P(NIPAM-co-MAA) microgels at various temperatures, illustrating the thermo-responsive volume phase transition. Scale bar: 1 μm.

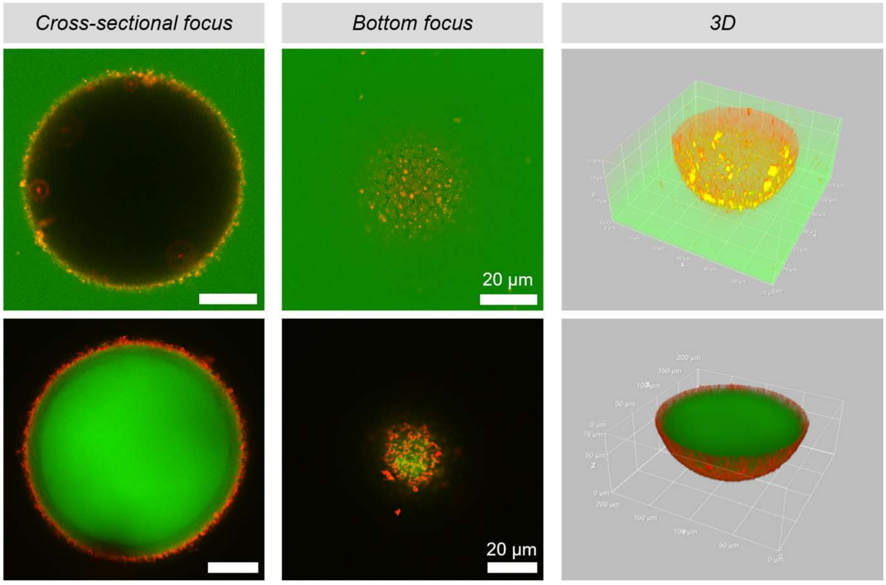

Figure S3. CLSM characterization of single O/W and W/O emulsion droplets. Confocal laser scanning microscopy (CLSM) images of a single O/W droplet (top) and a single W/O droplet (bottom) of Pickering emulsions, recorded at cross-sectional and bottom focal planes, together with their 3D reconstructed images. Scale bar: 20 μm.

Table S1. Droplet size statistics of emulsions stabilized by S3 hybrid microgels at different temperatures (4–50 °C) and pH conditions.

Temperature (°C)	pH	Mean ECD ± SD (μm)	CV	Mean AR ± SD	Type
4	2	142.7 ± 47.4	0.33	1.42 ± 0.63	W/O
	4	103.1 ± 37.8	0.37	1.18 ± 0.26	O/W
	6	120.0 ± 46.6	0.39	1.16 ± 0.18	O/W
	8	85.4 ± 29.5	0.35	1.30 ± 0.51	O/W
35	2	111.1 ± 42.1	0.38	1.44 ± 0.45	W/O
	4	136.2 ± 45.1	0.33	1.31 ± 0.34	W/O
	6	132.9 ± 50.9	0.39	1.37 ± 0.40	W/O
	8	127.3 ± 48.8	0.38	1.39 ± 0.53	W/O
50	2	106.5 ± 31.3	0.29	1.36 ± 0.42	W/O
	4	100.0 ± 28.0	0.28	1.26 ± 0.36	W/O
	6	116.3 ± 51.0	0.44	1.38 ± 0.43	W/O
	8	112.9 ± 50.4	0.45	1.53 ± 0.49	W/O

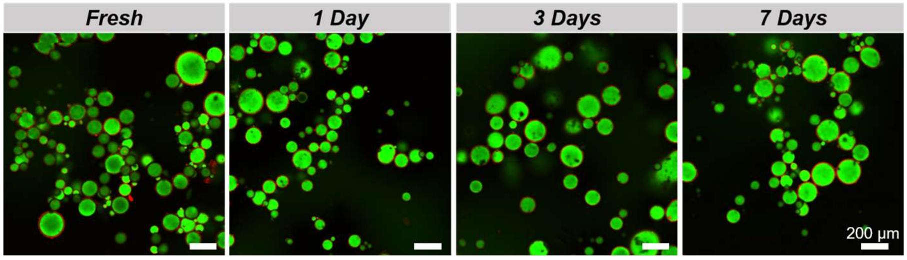

Figure S4. Stability of W/O Pickering emulsions stabilized by hybrid microgels. CLSM images of W/O Pickering emulsions stabilized by H-SiO_2@P(NIPAM-co-MAA) (S3) at 25 °C with an oil–water ratio of 2:1 and pH 7, recorded immediately after preparation, and after 1, 3, and 7 days of storage. Scale bar: 200 μm.
